# A review of existing neonatal hyperbilirubinemia guidelines in Indonesia

**DOI:** 10.12688/f1000research.110550.2

**Published:** 2023-11-13

**Authors:** Mahendra Tri Arif Sampurna, Danny Chandra Pratama, Visuddho Visuddho, Novita Oktaviana, Achmad Januar Er Putra, Rahmi Zakiyah, Jordy Maulana Ahmad, Risa Etika, Kartika Darma Handayani, Martono Tri Utomo, Dina Angelica, Wurry Ayuningtyas, Toto Wisnu Hendrarto, Rinawati Rohsiswatmo, Setya Wandita, Risma Karina Kaban, Kian Djien Liem

**Affiliations:** 1 Neonatology Division, Department of Pediatrics, Dr Soetomo General Hospital, Faculty of Medicine, Universitas Airlangga, Surbaya, 60115, Indonesia; 2 Department of Pediatrics, Airlangga University Teaching Hospital, Faculty of Medicine, Universitas Airlangga, Surabaya, 60115, Indonesia; 3 Medical Program, Faculty of Medicine, Universitas Airlangga, Surbaya, 60115, Indonesia; 4 Neonatal Intensive Care Unit, Harapan Kita Mother and Child Hospital, Jakarta, 11420, Indonesia; 5 Neonatology Division, Department of Pediatrics, Cipto Mangunkusumo Hospital, Faculty of Medicine, Universitas Indonesia, Jakarta, 10430, Indonesia; 6 Neonatology Division, Department of Child Health, Faculty of Medicine Public Health and Nursing, Universitas Gadjah Mada, Yogyakarta, 55281, Indonesia; 7 Department of Neonatology, Radboud University Medical Centre, Nijmegen, 6525, Netherlands Antilles

**Keywords:** icterus, neonates, recommendations, LMIC

## Abstract

**Background:**

Neonatal hyperbilirubinemia is one of the most common conditions for neonate inpatients. Indonesia faces a major challenge in which different guidelines regarding the management of this condition were present. This study aimed to compare the existing guidelines regarding prevention, diagnosis, treatment and monitoring in order to create the best recommendation for a new hyperbilirubinemia guideline in Indonesia.

**Methods:**

Through an earlier survey regarding adherence to the neonatal hyperbilirubinemia guideline, we identified that three main guidelines are being used in Indonesia. These were developed by the Indonesian Pediatric Society (IPS), the Ministry of Health (MoH), and World Health Organization (WHO). In this study, we compared factors such as prevention, monitoring, methods for identifying, risk factors in the development of neonatal jaundice, risk factors that increase brain damage, and intervention treatment threshold in the existing guidelines to determine the best recommendations for a new guideline.

**Results:**

The MoH and WHO guidelines allow screening and treatment of hyperbilirubinemia based on visual examination (VE) only. Compared with the MoH and WHO guidelines, risk assessment is comprehensively discussed in the IPS guideline. The MoH guideline recommends further examination of an icteric baby to ensure that the mother has enough milk without measuring the bilirubin level. The MoH guideline recommends referring the baby when it looks yellow on the soles and palms. The WHO and IPS guidelines recommend combining VE with an objective measurement of transcutaneous or serum bilirubin. The threshold to begin phototherapy in the WHO guideline is lower than the IPS guideline while the exchange transfusion threshold in both guidelines are comparably equal.

**Conclusions:**

The MoH guideline is outdated. MoH and IPS guidelines are causing differences in approaches to the management hyperbilirubinemia. A new, uniform guideline is required.

## Abbreviations

IPS: Indonesian Pediatric Society

MoH: Ministry of Health

WHO: World Health Organization

TSB: Total Serum Bilirubin

VE: Visual Examination

LMIC: Low Middle Income Countries

## Introduction

Neonatal hyperbilirubinemia is mostly discovered in the first week of life, both in a term infants (≥ 50%) and in preterm infants (≥ 80%). ^1^ The prevalence of severe neonatal jaundice in Indonesia according to the 2015 Don Ostrow Trieste Yellow Retreat was 6.8%, whereas acute bilirubin encephalopathy was 2.2%, leading to a Case Fatality Rate (CFR) caused by severe jaundice and acute bilirubin encephalopathy (ABE) of 24.2% and 74.9%, respectively. ^2^ However, most of the deaths from ABE were associated with neonatal sepsis. ^2^

Neonatal hyperbilirubinemia is often considered to be a threat both by doctors and families, so clear guidelines are needed to avoid overtreatment or under diagnosis. The prevalence of neonatal hyperbilirubinemia and kern-icterus is still high in Indonesia. ^2^ This may be due to a lack of awareness or adherence to existing guidelines, and there are also variations in the management of neonatal hyperbilirubinemia in Indonesia. ^3^

In Indonesia, there are three main guidelines on the management of neonatal hyperbilirubinemia. ^3^ This literature review intends to explore the three guidelines that have been used in Indonesia to seek recommendations for the management of neonatal hyperbilirubinemia. These guidelines are used by various health care practitioners, although it has been found that the MoH guideline is widely used by midwives and nurses, the WHO guideline is widely used by general practitioners, and the IPS by pediatricians. ^3^

The existing guidelines have their weaknesses. The authors perspectives on Ministry of Health guidelines are that they are irrelevant with current evidence based on medical knowledge nowadays and that the WHO guidelines are considered unsuitable for use in Indonesia because they are intended for countries with limited facilities and do not yet have national guidelines. The IPS guidelines, which refer to the American Academy of Pediatrics (AAP), are not fully applicable in Indonesia due to the inadequacies of health facilities in Indonesia. ^4^^,^^5^ According to a preliminary survey in Indonesia, the IPS guideline is difficult to access by 50% of pediatricians. ^3^

It is necessary to have uniform national guidelines regarding management of neonatal hyperbilirubinemia which can overcome the pitfalls of each existing guideline. This review will examine the differences in the recommendations of various neonatal hyperbilirubinemia guidelines and assess whether they are in accordance with the latest evidence.

## Methods

### Source data

According to the previous survey, 84% of pediatricians adhered to Indonesia Pediatric Society (IPS) guidelines, 46% of general practitioners adhered to World Health Organization (WHO) guidelines and 46% midwives adhered to Ministry of Health (MoH) guidelines. ^3^ It was concluded that Indonesia’s healthcare workers uses three main guidelines: first, the IPS 2011 guideline in the Medical Service Guidelines, Second Edition; ^5^ second, the guideline released by the MoH in 2018; ^6^ and third, the guidelines recommended by WHO in 2013, published in the blue pocket book of hospital care for children. ^7^ Please see *Underlying data* for information on where these reports can be accessed.

We investigated each guideline to find out the differences between the three, to create a unity guideline for Indonesia.

### Data synthesis and quality assessment

All authors reviewed each recommendation of the guideline in terms of:

1. Prevention and monitoring of neonatal jaundice.
2. Methods for identifying neonatal hyperbilirubinemia.
3. Risk factors in the development of neonatal hyperbilirubinemia.
4. Risk factors that might increase the risk of brain damage at levels below the accepted level for neonatal hyperbilirubinemia.
5. Intervention treatment threshold.

We compared all of the aforementioned recommendations domain in each guideline and evaluated its applicability in Indonesia.

**Figure 1.**
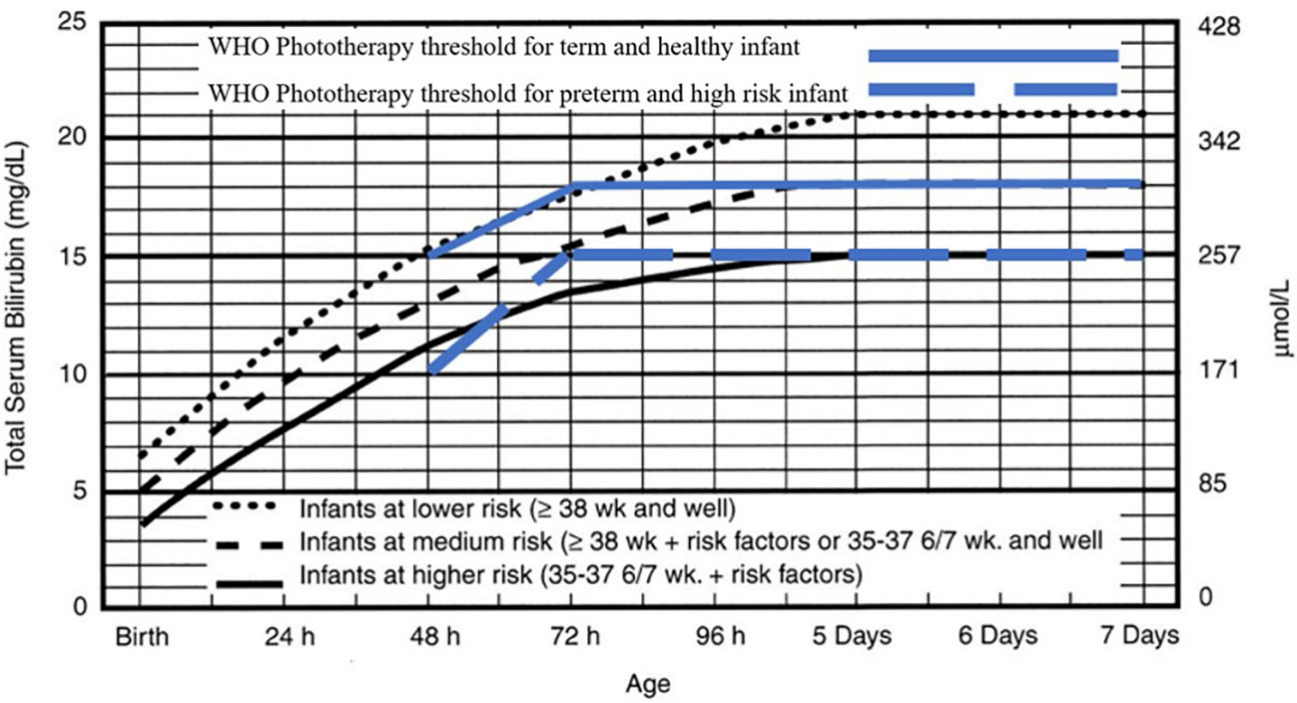
Phototherapy for infants ≥ 35 weeks' gestation. Indonesian Pediatric Society phototherapy threshold adopted from American Academy of Pediatrics. Management of hyperbilirubinemia in the newborn infant 35 or more weeks of gestation. ^8^ Reproduced with permission from Journal Pediatrics, Vol. 114, Page 20, Copyright © 2022 by the AAP.

**Figure 2.**
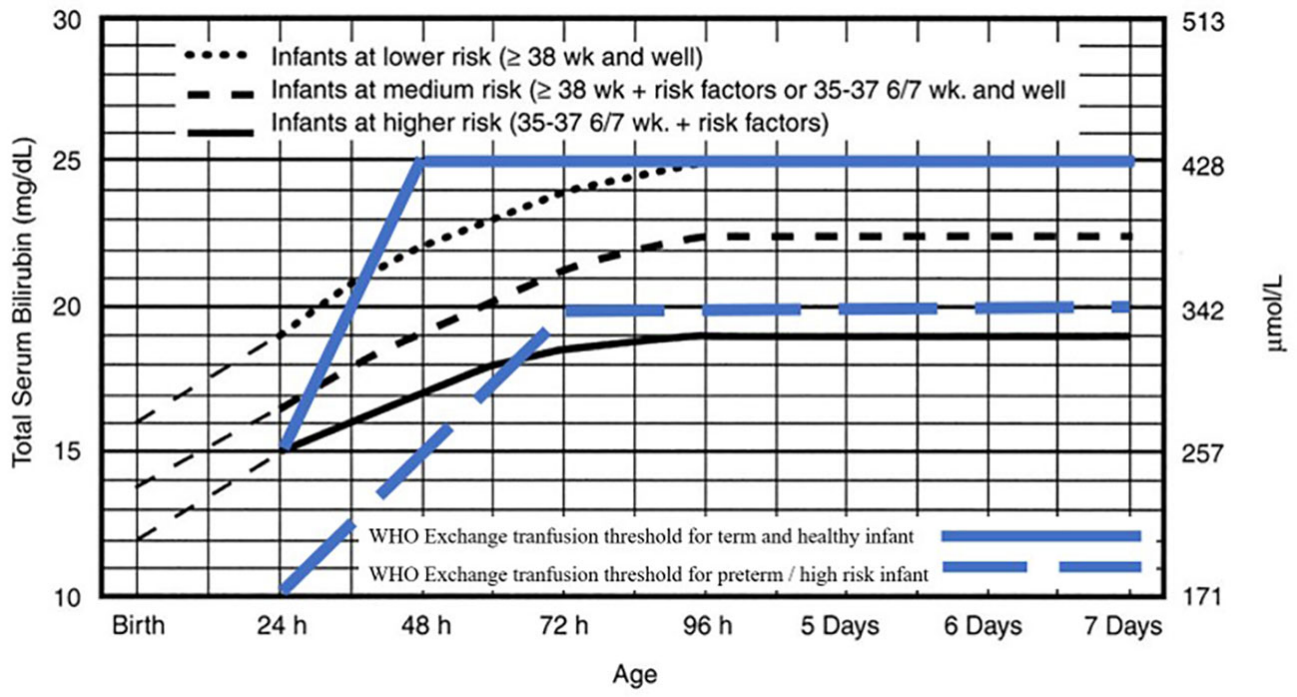
Exchange transfusion threshold for infants ≥ 35 weeks' gestation. Indonesian Pediatric Society phototherapy threshold adopted from American Academy of Pediatrics. Management of hyperbilirubinemia in the newborn infant 35 or more weeks of gestation. ^8^ Reproduced with permission from Journal Pediatrics, Vol. 114, Page 20, Copyright © 2022 by the AAP.

Two reviewers (MT and KD) collaborated on the quality assessment through a group discussion, and the final decision was made based on their agreement. The extracted data was summarized in a comparison table. The intervention treatment threshold was shown in the Figure 1 and Figure 2 for both normograms from IPS and WHO.

## Results

Recommendations regarding the monitoring of neonatal jaundice, methods for detecting hyperbilirubinemia, and risk factors for the development of neonatal hyperbilirubinemia were established in each of the guidelines. However, only the IPS and WHO guidelines mention risk factors for the development of brain injury at levels below the accepted levels for healthy term infants, the levels at which to start and stop phototherapy, and the levels at which to consider the exchange transfusion. The IPS guideline is mainly based on the American Academy of Pediatrics guideline for term and near-term babies of ≥ 35 weeks gestational age.

### Prevention and monitoring of jaundiced neonates

The IPS guidelines advise measuring the bilirubin level in infants at discharge and after discharge. They also state that jaundice should be assessed under natural light whenever vital signs of the infant are measured (in hospital) but does not mention a specific time. The IPS recommends measuring the total serum bilirubin when the baby is prepared for discharge, with the results to be plotted on a specific nomogram.

MoH guidelines advise using visual examination (VE) to classify jaundice neonates into three categories: no jaundice, jaundice, and severe jaundice.

The WHO guideline does not clearly mention how to monitor the jaundice step by step. The WHO suggests using visual estimations to differentiate normal from abnormal neonatal jaundice. Abnormal neonatal jaundice in the WHO guidelines is defined by the occurrence of jaundice in the first day of life, followed with a fever lasting > 14 days in term and > 21 days in pre-term; babies with deep jaundice (jaundice in the palms and soles) are considered to have abnormal jaundice when the babies are preterm and < 35 week of gestation. The definition and recommended actions are listed in Table 1.

**Table 1.** Set of recommendations for prevention and monitoring of neonatal hyperbilirubinemia. ^5^^–^^7^

IPS	MoH	WHO
Measure at discharge (before 72 hours) and risk categorization based on Bhutani Nomogram ^13^, plot to the specific nomogram from AAP ^4^. Find the possibility of excessive production of bilirubin and assess risk factors: increased enterohepatic circulation, defective uptake and conjugation, slow excretion. Pay attention to the sign of non-physiological jaundice: TSB treatment threshold, jaundice in the first day of life, increasing TSB > 5mg/dl/24 hours, direct bilirubin > 2mg/dL, prolonged jaundice > 2 weeks if the baby is ill (vomiting, poor feeding, dehydration, fever, losing-weight, apnea or distress) Follow up for the following 2 days after 72-hours of discharge	Uses VE and jaundice categorization: **Severe jaundice**: • Baby looks yellow in first day of life or > 14 days of life. • Yellow on palms and soles, yellow and pale stool. **Jaundice**: • Baby looks yellow but not on palms and soles. • Baby looks yellow at day 2–4 post-natal. **No Jaundice**: Primary neonatal care: Give breast milk and keep the baby warm and kangaroo mother care.	Use VE and jaundice categorization **Normal (physiological)** • Skin and eyes yellow but none of the signs of abnormal jaundice below. **Abnormal (non-physiological):** • Begin on the first day of life. • Lasting more than 14 days in term and 21 days in preterm infants. • Accompanied with fever. • Deep jaundice: palms and soles of the infant are deep yellow.

Note: IPS = Indonesian Pediatric Society; MoH = Ministry of Health; WHO = World Health Organization; AAP = American Academy of Pediatrics; TSB = Total Serum Bilirubin; VE = Visual Estimation.

### Monitoring

The IPS guideline recommends a follow-up clinic at a maximum of 2–4 days after discharge from hospital. ^5^ According to MoH guidelines, 2019, monitoring for jaundiced babies is one day after discharge from health facilities. A general assessment to determine severe jaundice/jaundice/no jaundice is required to decide the next step of recommendation. The WHO does not a mention specific age for the follow-up outpatient clinic. The WHO recommends that all newborns should be monitored for the development of jaundice, which should be confirmed by a bilirubin measurement, when possible, in cases where jaundice appears on day one in preterm infants (< 35 weeks), and where jaundice appears on day two for infants whose palms and soles are yellow at any age. The set of recommendations for the causes of hyperbilirubinemia are listed in Table 2.

**Table 2.** Set of recommendations to seek the cause of neonatal hyperbilirubinemia. ^5^^–^^7^

IPS	MoH	WHO
1. Monitor of history of jaundice in the family. 2. History family of anemia, splenectomy, spherocytosis, glucose 6-fosfat --dehydrogenase deficiency(G6PD). 3. Family history of liver disease: galactosemia, alfa-1-antitrypsin-deficiency, tyrosinosis, hypermethioninemia, Gilbert syndrome, Crigler-Najjar syndrome, or cystic fibrosis. 4. ABO/Rhesus incompatility. 5. Congenital infection: toxoplasmosis, cytomegaloviruses. 6. Maternal drugs: sulfonamide, nitrofurantoin, antimalarial. 7. Birth trauma: asphyxia, intracranial hemorrhage, cephalohematoma. 8. Delayed cord clamping resulting in polycythemia. 9. Total parenteral nutrition.	1. When a baby is diagnosed with severe jaundice: the guideline recommends to immediately refer the baby to better service facilities and keep maintaining blood sugar and temperature. 2. When the baby is diagnosed with jaundice: encourage the mother to give more breast milk and advise her on when to return, preferably the following day. Monitor the signs of dehydration: infant urination should be (≥ 6 times a day) and defecation more than twice a day. Teach the mother to nurture the baby and return the following day.	Search for the possibility of: 1. Serious bacterial infection. 2. Hemolytic disease due to blood group incompatibility or glucose 6-phosphate dehydrogenase deficiency. 3. Congenital syphilis or other intrauterine infection. 4. Liver disease such as hepatitis or biliary atresia (stools pale and urine dark). 5 Hypothyroidism. The investigations depend on the probable diagnosis and what tests are available, but may include: 1. Hb or packed cell volume. 2. Complete blood count to identify signs of serious bacterial infection (high or low neutrophil count with > 20% band forms) and signs of hemolysis. 3. Blood type of infant and mother, and Coombs test. 4. Syphilis serology, such as VDRL tests. 5. Glucose 6-phosphate dehydrogenase screening, thyroid function tests, liver ultrasound.

Note: IPS = Indonesian Pediatric Society; MoH = Ministry of Health; WHO = World Health Organization; TSB = Total Serum Bilirubin.

### Methods to identify neonatal hyperbilirubinemia

The IPS and WHO guidelines recommend using visual inspection in combination with objective measurement, for example Transcutaneous Bilirubin or Total Serum Bilirubin. ^5^^,^^7^ The WHO recommends all newborns should be monitored for the development of neonatal jaundice, which should be confirmed by a bilirubin measurement, if the facility is available. ^7^ The guideline of the MoH recommends solely visual inspection as the method to detect neonatal hyperbilirubinemia. When infants look very yellow (in palms and soles), the MoH recommends immediate referral to higher level health facilities for further examination. ^6^

### Risk factor for the development of neonatal hyperbilirubinemia

We found a rather large variation in the guidelines on factors that may increase the risk of the development of neonatal hyperbilirubinemia. Only prematurity and hemolytic diseases are recognized as risk factors for developing neonatal hyperbilirubinemia in all guidelines. Otherwise, each guideline has a different set of risk factors that might increase the risk of developing neonatal hyperbilirubinemia. The risk factors for hyperbilirubinemia are listed in Table 3.

**Table 3.** Risk factors of hyperbilirubinemia. ^5^^–^^7^

IPS	MoH	WHO
Major risk factors: • A total serum bilirubin levels before discharge in the high-risk zone (based on Bhutani nomogram). • Icterus occurs in the first 24 hours, blood group incompatibility with positive antiglobulin testing or other hemolytic diseases (for example, glucose 6-phosphate dehydrogenase (G6PD deficiency). • 35–36 weeks’ gestation. • History of family receiving phototherapy. • Cephalohematoma or extensive bruising. • Exclusive breastfeeding, especially with inadequate dietary intake and excessive weight loss. • East Asian race. Minor risk factors: • Total serum bilirubin levels before discharge from hospital in the intermediate risk zone. (based on Bhutani nomogram) • 37–38 weeks' gestation. • Icterus occurs before discharge. • Family history of jaundice. • Macrosomia baby from gestational diabetic mothers.	• Yellow in first 24 hours. • Severe bacterial infection. • Premature babies. • Low birth weight. • Problems with breastfeeding. • Birth trauma (ecchymosis and hematoma).	• Severe bacterial infections. • Hemolytic disease (blood group incompatibility and glucose 6-phosphate dehydrogenase deficiency (G6PD deficiency). • Congenital syphilis or other intrauterine infections, liver disease (hepatitis or biliary atresia). • Hypothyroidism.

Note: IPS = Indonesian Pediatric Society; MoH = Ministry of Health; WHO = World Health Organization.

### Risk factors that might increase the risk of brain damage at levels below the accepted level for neonatal hyperbilirubinemia

In all guidelines, factors are mentioned that might increase the risk for bilirubin encephalopathy at levels below the risk level in healthy infants. ^5^^–^^7^At the same time, there is a wide variation in the included risk factors. Although the MoH guideline does not identify the risk factors, it does mention severe bacterial infection, which is also included in other guidelines. ^6^ The other risk factors included in the WHO and IPS guidelines is severe hemolysis. ^5^^,^^7^ Birth asphyxia is not stated in the MoH and WHO guidelines. ^6^^,^^7^

Only the IPS guideline mentions in detail other risk factors of brain damage that are below accepted levels for neonatal hyperbilirubinemia, such as if the babies are sick or have had asphyxia, hypoalbuminemia < 3.0 mg/dL or prolonged acidosis. ^4^^,^^5^

### Intervention treatment threshold

The IPS guideline, widely used by pediatricians, recommends nomogram for phototherapy and exchange transfusion for infants with a gestational age of ≥ 35 weeks. The phototherapy threshold in the IPS guideline rises then plateaus at five days of postnatal age. The IPS distinguished the level of the baby into three categories, lower risk, medium risk, and higher risk. At the age of 24 hours, the thresholds are 12mg/dL, 10mg/dL, and 8mg/dL, respectively. At 48 hours, they are 15mg/dL,13mg/dL, and 11mg/dL, respectively. At 72 hours, they are 18mg/dL,15 mg/dL, and 13mg/dL, respectively, and at 96 hours, they are 20mg/dL, 17mg/dL, and 14mg/dL, respectively. Meanwhile, at five days they are 21mg/dL, 18mg/dL, and 15mg/dL, respectively, and then plateau. ^5^ These thresholds are referred to in Figure 1.

The 2013 WHO guideline states that the management of neonatal jaundice with phototherapy and exchange transfusion is based on whether the infant is term or preterm, the age of the infant with hyperbilirubinemia, and the total serum bilirubin. The WHO also mentions that phototherapy can be given if babies have jaundice on the first day of life, as well as for icteric premature babies, icteric babies due to hemolysis, and babies who have severe jaundice (yellow on the palms of the hands and feet). The WHO recommends measuring Total Serum Bilirubin (TSB) if a laboratory is available and recommends referring to the table as cut off value for healthy babies of > 35 weeks’ gestation or < 35 weeks (higher risk infants) of gestation without mentioning the birth weight of the baby. At 48 hours postnatally, the WHO recommends starting phototherapy at 15mg/dL and 10mg/dL for healthy and higher risk infants, respectively. At 72 hours postnatally, it recommends starting with 18mg/dL and 15mg/dL, respectively ( Figure 1). ^6^

The exchange transfusion process is not described in the WHO guidelines. The recommendation, shown in Figure 2, of the serum bilirubin levels threshold for an exchange transfusion can only be effective if the infant can be transferred quickly and safely to another facility where exchange transfusion can be performed. ^6^ The risk factors reported in the WHO guideline are illustrated in the figure and include small size (< 2.5kg at birth or born before 37 weeks’ gestation), hemolysis and sepsis. ^6^ Both the IPS and WHO guidelines use 25mg/dL as a threshold for healthy term infants and around 20mg/dL for higher risk infants ( Figure 2). ^4^^,^^6^

The MoH guidelines do not include an intervention treatment threshold because the guidelines used in primary healthcare settings and the guidelines recommend to refer the baby when it looks yellow on the soles and palms (severe jaundice).

## Discussion

We identified important differences in the existing guidelines on neonatal hyperbilirubinemia in Indonesia. We detected differences in the monitoring of infants in the first week of life, in the sets of recommendations for seeking the cause of neonatal hyperbilirubinemia, in the methods to detect neonatal hyperbilirubinemia, in the inclusion of risk factors for both the development of neonatal hyperbilirubinemia and an increased brain vulnerability, and in the advice for when to start phototherapy and exchange transfusion.

### Prevention and monitoring of neonatal jaundice

Assessing yellow babies by eye is an inevitable practice because it is fast and easy and does not require additional instruments that require investment. ^8^ Unfortunately, the guidelines in Indonesia do not clearly identify the steps required to recognize a yellow baby by eye and how to interpret the results when using this method. In fact, the Ministry of Health guideline only uses visual estimates in determining severity based on the level of yellowness, with the added recommendation of referring the yellow baby if it is already yellow through the palms and sole. This could put the baby at risk. The procedure for assessing a yellow baby should be explained in detail and include using natural lighting and pressing and pulling the skin from both sides, starting from the head, chest, abdomen, groin, palms, and soles. ^9^ Delays in diagnosing and treating the case are thought to be the cause of the high incidence of severe neonatal hyperbilirubinemia and kernicterus because a study confirmed that visual examination has a high interobserver variability resulting in low and variable TSB results, which in turn will have an impact on inappropriate diagnosis and treatment. ^10^^–^^12^ The WHO guideline, when referring to developed countries that do not have any neonatal hyperbilirubinemia guidelines, recommends performing phototherapy in yellow baby cases within the first 24 hours, in premature yellow babies, in babies with yellowish color through the palms and soles, and in yellow babies who have not had a bilirubin examination. In contrast, the WHO guideline recommends measuring total serum bilirubin if jaundice appears on day one, for preterm infants (< 35 weeks) if jaundice appears on day two, and for infants whose palms and soles are yellow at any age. However, the IPS guidelines recommend a TSB examination and plotting on a nomogram according to the baby risk category and postnatal age in hours. In the field, this could potentially create confusion in the management of yellow babies. ^3^

The IPS recommends conducting a risk factor assessment using the Bhutani nomogram curve to determine the risk category for an infant developing severe neonatal hyperbilirubinemia. This is in accordance with the recommendations from AAP. Somehow only half of the pediatricians who intend to use this guideline are able to implement this recommendation, although pre-discharge risk assessment is important to evaluate neonatal hyperbilirubinemia. ^3^^,^^13^ Pre-discharge serum bilirubin was plotted on hour-specific percentile charts (“nomograms”) and accurately predicted impending neonatal hyperbilirubinemia, according to the findings of two studies. ^8^^,^^14^ A study revealed a remarkable improvement in the incidence of neonatal hyperbilirubinemia due to the application of the screening program. The proportion of infants with serum bilirubin levels ≥ 342 micromol/L (3.87 mg/dL) decreased from one in 77 to one in 142 ( *P* < 0.0001), while the proportion of infants with serum bilirubin levels ≥ 427 micromol/liter (4.83 mg/dL) decreased from one in 1522 to one in 4037 ( *P* < 0.005), and the rate of hospital readmissions for neonatal hyperbilirubinemia decreased dramatically from 5.5 per 1000 infants before the program to 4.3 per 1000 infants after its implementation ( *P* < 0.005). ^15^ We discovered that a universal screening program along with an assessment of bilirubin using a percentile-based nomogram could decrease the occurrence of neonatal hyperbilirubinemia and hospital readmissions for phototherapy.

However, the method for identifying bilirubin levels is still challenging for LMICs (Low Middle Income Countries) such as Indonesia.

The availability of TcB (Transcutaneous Bilirubin), and laboratory facilities has had a great impact on the implementation of this recommendation, and it will be necessary to conduct research in Indonesia in this area. There is a similarity between the IPS, the MoH and the WHO regarding the identification of jaundice in the first 24 hours of birth, regardless of its severity and if a baby appears unwell. It is important to monitor serum bilirubin levels in the first 24 hours of life (serum bilirubin–day 1). ^4^^–^^6^ Evidence from Carbonel *et al*., Agarwal *et al*., and Alpay *et al*. showed that serum bilirubin ≥ 102 micromol/liter (6mg/dL) on day one is a sensitive predictor of serum bilirubin > 290 micromol/liter (17mg/dL) between days three and five. ^16^^–^^18^

The Neonatal Institute for Health and Care Excellence (NICE) guideline recommends that visible jaundice in the first 24 hours is an important predictor of neonatal hyperbilirubinemia. ^19^ Any visible or suspected jaundice in the first 24 hours requires urgent medical assessment within two hours, including serum bilirubin measurement and an investigation of the underlying causes. ^20^

The IPS emphasizes a more objective examination as an additional measurement by TSB. This examination could be used to monitor jaundice if the level of TSB increases > 5 mg/dL/24 hours or cholestasis alertness if direct bilirubin increases > 2 mg/dL, whereas the MoH and WHO guidelines only give a warning if jaundice occurs in > 14 days and if there are pale stools. These are very likely because the MoH and WHO guidelines are mostly used by midwives and general practitioners who mostly work in primary health care facilities with limited equipment, while the IPS is used by 80% of pediatricians working in referral facilities. ^3^ The WHO also uses a set of recommended risk factors for developing abnormal neonatal jaundice similar to the IPS, such as suspected hemolysis, syphilis, intrauterine infection, and liver disease, which may only be detected at a referral hospital. These overlapping factors will create confusion in the handling of a jaundiced baby. In addition, this set of recommendations might be difficult to establish in a constrained setting where the WHO guidelines are implemented. A uniform set of recommendations will make it easier for health workers to use the guidelines.

### Methods to identify neonatal hyperbilirubinemia

The MoH guidelines do not mention a method for identifying a yellow baby other than VE. The MoH only recommends immediate referral to hospitals that have complete laboratory facilities. This indicates that this guideline is only intended for use in primary health care, which could be dangerous, as diagnosing neonatal jaundice only using degrees of yellow on the palms and soles might cause delays in the identification process.

A significant false-negative rate was also discovered in a study when visual assessment was used. There was a high rate of clinical misclassification, with 61.5% (67 of 109) of infants whose serum bilirubin was in the high-risk zone being incorrectly placed in the low-risk zone. In addition, 8.1% (230 out of 2857) of infants whose clinical estimation was placed in the low risk zone had serum bilirubin values in the higher risk zones. ^12^

Inconsistent findings were found on the diagnostic accuracy of visual examination in determining the severity of jaundice. One study found that the 'caudal to nipple line' method of assessing jaundice was 97% sensitive and 19% specific for detecting serum bilirubin levels > 205 micromol/liter (12 mg/dL), whereas another found sensitivity of 76% and specificity of 60%, and both reported that cephalo-caudal progression is more accessible to parents than health care workers. ^11^^,^^21^^,^^22^

NICE recommends visual examinations for all babies to check whether there are risk factors with an increased probability of developing significant hyperbilirubinemia soon after birth by examining a jaundiced baby, especially in the first 72 hours. Parents, carers and healthcare professionals should all look for jaundice (visual inspection). Visual examinations of a naked baby should be done in bright and preferably natural light. Examination of the sclerae, gums and blanched skin is applicable in all skin colors and also ensures that babies with a risk factor for developing significant neonatal hyperbilirubinemia receive an additional visual inspection by a healthcare professional during the first 48 hours of life. ^19^ NICE guidelines recommend not relying on visual inspection alone to estimate the bilirubin level in a baby with jaundice. The bilirubin level should be measured and recorded within six hours in all babies more than 24 hours old with suspected or obvious jaundice. ^19^

The WHO does not necessarily recommend diagnosing yellow babies based on laboratory examinations in every case of jaundice, and it depends on the availability of laboratory facilities. ^6^ The WHO recommends testing the serum bilirubin of jaundice occurring in the first 24 hours of life, in babies with yellow palms and soles, and in premature infants that are yellow and adhere to the MoH guidelines for severe jaundice. ^5^^,^^6^ If this protocol is routinely undertaken, severe neonatal hyperbilirubinemia can be prevented by monitoring serum bilirubin levels in infants who appear jaundiced and follow the therapeutic threshold according to the IPS guidelines, which cannot be avoided if health workers are still using and adhering to the WHO and MoH guidelines. The scarcity of laboratory resources and access to laboratory tests for serum bilirubin and risk identification in low- and middle-income countries has become another problem in the application of the IPS guidelines in various areas with different facilities. ^2^

To overcome these problems, there are several methods for diagnosing neonatal hyperbilirubinemia that are less invasive, available worldwide, and have lower investment costs. These include TcB (Transcutaneous Bilirubin), Billistick (POCT), and the smartphone app (Billicam, Picterus). ^23^^–^^25^ TcB is excellent for fast and reliable estimation and is non-invasive, meaning it can reduce the need for blood sampling; However, TcB is expensive, needs daily calibration and regular maintenance, and unfortunately is not sufficiently accurate in infants treated with phototherapy due to skin bleaching **.** ^25^ Transcutaneous bilirubin measurement could reduce the frequency of hospital re-admissions, and its routine use may lead to a reduction in the number of blood samples collected for bilirubin estimation, but it has an associated increase in the use of phototherapy. ^26^^–^^28^ A study showed a reduction of 55% in blood sampling was reported if serum bilirubin testing was limited to babies with transcutaneous bilirubin levels > 195 micromol/liter, whereas another study in 285 healthy babies at > 34 weeks of gestation showed a reduction of 34% in the number of blood samples taken. ^29^^,^^30^ A retrospective analysis showed that 35% (178 of 504) of the NICU babies and 80% (254 of 317) of the healthy term and near-term babies would have avoided blood sampling for serum bilirubin estimation. Some investigation in this non-invasive device is mandatory. ^31^

Meanwhile, with its pervasiveness, portability, and low cost, the smartphone shows potential advantages for screening in home environments where TcBs are not available, and its user-friendly design ensures that anyone can use the app after watching the short tutorial video. However, it has limitations when it comes to different brands and models, which employ a variety of cameras, lenses, filters, and color corrections that could impact data results. The reliability of smartphone- based screening in dark pigmented skin is rather low compared with Caucasian skin. ^23^ POCT (Bilistick) can be used in infants treated with phototherapy, is low cost, and only needs a small amount of blood, so it is very useful in low- to middle-income countries, where access to expensive laboratories may be limited. This method is still under development. ^2^^,^^23^^,^^24^^,^^32^^–^^34^

In conclusion, TcB still represents the most reliable solution. The IPS guideline has indeed recommended the use of TcB, but in Indonesia there are only about 16 hospitals that use it, which may be due to the costs involved. The interpretation and use of TcB is not explained in the guidelines and is not so well known: only one out of five academic teaching hospitals in Indonesia are using TcB. ^35^

### Risk factors for the development of neonatal hyperbilirubinemia

The risk factors listed in the IPS guideline are more complete, clear, and sequential than the risk factors listed in the other two guidelines, and IPS guideline pays special attention to areas that have a high prevalence of neonatal hyperbilirubinemia cases. This is very helpful in early detection and is expected to reduce the incidence of neonatal hyperbilirubinemia. Of the three guidelines, there is some mention the same risk factors, but in the IPS guidelines, these risk factors are mentioned more clearly, sequentially and more completely. There are some difficulties in analyzing the risk factors in the MoH guidelines because several risk factors are listed in different sections, so it is necessary to read the guidelines carefully.

There are four factors that are independently associated with an increased risk of neonatal hyperbilirubinemia based on the NICE guideline: a gestational age of < 38 weeks, jaundice within 24 hours of birth, intention to breastfeed exclusively, and previous history of a sibling with neonatal jaundice. ^14^^,^^19^^,^^36^^–^^40^ The MoH guideline places greater emphasis on analysis and dehydration assessment as a result of inadequate lactation management. The parameters of defecation and urination frequency are very important in the MoH guideline. If we use clinical parameters, it is better to use weight scaling every day, which is likely to be applied in primary health care. Studies show that > 10%–12% weight loss will provide a better assessment of the degree of dehydration, and is a predictor of infants turning yellow later in life. ^41^ Anamnesis about breastfeeding techniques and the adequacy of breast milk can be recommended, including the number of feeds per day; an assessment of the attachment position of breastfeeding; an assessment of the breastfeeding process and of the condition of the baby; consideration of factors that affect breastfeeding success, such as tongue tie and blisters on the breast; history of the condition of the baby, such as the presence of fever; and the comforting of the infant between feeds, which will complete the clinical parameters in assessing the success of breastfeeding. The steps for assessing and examining the blood glucose in the MoH are very well done, but recommendations for adequate infant fluid intake requirements should be added to the MoH guidelines to prevent the jaundice from worsening.

A study was conducted to examine the association between jaundice noted in the first 24 hours of life, and the risk of later neonatal hyperbilirubinemia and the need for phototherapy. The early jaundiced babies were found to have a statistically significant increase in the risk of developing neonatal hyperbilirubinemia above 427 micromol/liter (25mg/dL), and these babies were ten times more likely to be treated with phototherapy compared with newborns noted not to have jaundice in the first 24 hours (OR 10.1, 95% CI 4.2 to 24.4). ^20^ A prospective cohort study evaluated the early serum bilirubin measurements to predict neonatal hyperbilirubinemia in healthy term babies. Serum bilirubin levels of > 85 micromol/liter (5mg/dL) on day one had a statistically significant association with neonatal hyperbilirubinemia (adjusted OR 36.5, 95% CI 15.9 to 83.6). ^42^

A study showed clear trends that newborns who had one or more prior siblings with neonatal hyperbilirubinemia had a three-fold higher risk of developing neonatal hyperbilirubinemia compared with those who did not. There was a 2.7 times higher risk of mild jaundice in newborns who had a sibling with mild neonatal jaundice (OR 2.7, 95% CI 1.8 to 4.1), and the risk was four times greater for the moderate neonatal jaundice group (OR 4.1, 95% CI 1.5 to 10.8). The risk of developing jaundice for babies who had a prior sibling with severe neonatal hyperbilirubinemia was 12 times higher compared with those who had no sibling with severe neonatal hyperbilirubinemia (OR 12.5, 95% CI 2.3 to 65.3). ^38^^,^^42^

Prematurity is seen as a risk factor in all guidelines. This is due to the existence of low bilirubin-level kernicterus among preterm neonates. ^43^ Other problems often occurred with prematurity, such as hypoalbuminemia, inflammation/infection, and co-morbid central nervous system (CNS) findings, which synergistically act as contributing factors for bilirubin neurotoxicity. ^44^ It is not clear to us why almost every guideline has its own list of risk factors. There are good arguments for all of the risk factors.

The same variation in risk factors that might cause bilirubin to be toxic at lower levels in healthy infants was found. Severe hemolysis, and severe bacterial infection were the only factors mentioned. Asphyxia as a risk factor is only found in the IPS guidelines, and is one of the risk factors that plays a role in encephalopathy at low levels of hyperbilirubinemia, but it is not stated in the WHO and MoH guidelines. The MoH stated that asphyxia neonatorum was the main cause of neonatal death in Indonesia, and the WHO specifically explains how to deal with it. There is no clear explanation as to why asphyxia is considered as a risk factor in the IPS, but not in other guidelines. This could be because the two guidelines are aimed more at primary health care settings, and the recommendation is to immediately refer the baby when you find the risk factors above and also it might be due to the difficulty of diagnosing asphyxia in areas with limited resources and may be significantly different in a well-equipped area, so it is not a reflection of the real definition of asphyxia, which is metabolic acidosis and hypoxia, and the variable risk factors and susceptibility of bilirubin encephalopathy. MoH and WHO guidelines are intended for the low- and middle-income countries where asphyxia is common, accounting for as many as 814,000 neonatal deaths and 1.02 million still births, which is higher than in high income countries. Almost all of these deaths are in low- and middle-income countries, where women frequently cannot access quality perinatal care and may delay the seeking of care. Low- to middle- income countries with limited resources make it more difficult to diagnose asphyxia. ^45^^,^^46^ In the Netherlands, where a uniform guideline was developed, asphyxia is regarded as a risk factor because it had been reported in eight out of 10 health centers. ^47^

The WHO and MoH guidelines state that the causes of abnormal jaundice, such as severe infection and hemolysis, which are in the IPS guideline, play a role in causing bilirubin encephalopathy at lower bilirubin levels.

### Intervention treatment threshold

The IPS guideline has a lower threshold than the WHO guideline, so it is suitable for low- to middle-income countries and has a simpler graph (plateau on the third day) when compared to the IPS guideline (plateau on the fifth day). This difference in threshold is causing confusion in health workers in Indonesia because there is not any reason for the difference in the threshold, even though the WHO category of healthy babies uses a threshold of up to 2–3 milligrams lower than AAP for the infants at lower risk at > 38 weeks and that are healthy, but when conditions plateau on day five, the threshold was the same as for a medium risk baby (> 38 weeks with a risk factor, or a healthy 35–37 week baby) at 18mg/dL. Meanwhile, the WHO category of babies at risk has the same threshold as the IPS infants at high risk, which is limited to 15mg/dL. Whereas for the exchange transfusion guidelines, it seems that the WHO category of healthy infants has the same threshold of 25mg/dL after the plateau on the third day, which is the same level as the IPS guideline for the category of infants with the low risk category (> 38 weeks and healthy). Meanwhile, when compared to babies with the WHO risk, it has the same threshold as the IPS infants at high risk, which is around 20mg/dL. It seems that the WHO has made threshold guidelines simpler and more practical when compared to the IPS. According to the WHO guidelines, the threshold of infants requiring lower phototherapy may be intended to prevent severe hyperbilirubinemia in low- to middle-income countries with limited facilities, given the difficulty in identifying risk factors for hyperbilirubinemia and maintenance of phototherapy devices. ^46^^,^^48^

The MoH guideline is slightly different compared to the two previous guidelines. This guideline does not mention the threshold to start phototherapy or exchange transfusion, so it is more practical in areas that do not have phototherapy equipment, but if we would like to have a uniform guideline, there must be a link to unite the guidelines.

Additionally, a new AAP guideline was reinforced in 2022, recommending blood group and direct antiglobulin tests for all babies born to Rh-negative mothers with unknown antibody status. ^49^ This supports the identification of risk factors related to blood groups, as mentioned in the IPS and WHO guidelines but not in the MoH guideline. The guidelines also provide recommendations for when to check rebound bilirubin levels after phototherapy. While this recommendation aligns with the IPS guidelines, it differs from the MoH and WHO guidelines. However, none of the guidelines mention that follow-up can be conducted using TcB at least 24 hours after phototherapy. ^49^

Due to the new guideline’s slightly higher threshold, it is necessary to discontinue phototherapy when bilirubin levels are 2 mg/dL below the initial threshold and consider escalating care when they fall 2 mg/dL below the exchange transfusion threshold. These providing healthcare providers with increased confidence in using less phototherapy. This decision should be made after considering the neurotoxic risk threshold graph when assessing the need for treatment. Furthermore, the new guideline emphasizes the importance of reducing the likelihood of requiring phototherapy by promoting early and frequent feeding (8-12 times in 24 hours). ^49^ Taking into consideration a newly published guideline in 2022 may further support the necessity for a new uniform guideline.

A good guideline is uniform and needs to be connected to other guidelines. Although the MoH guideline for primary health care settings is only for screening, at least the referral recommendations include what actions should be taken to assist the baby. Hopefully health workers will understand the procedure so they can educate the family of the patient.

Based on our findings we recommend developing a new uniform guideline from the useful components from the three mentioned guidelines.

## Conclusions

We compared three existing guidelines for neonatal hyperbilirubinemia in Indonesia. The MoH guidelines tend to use clinical parameters to monitor the prevention of neonatal jaundice but are likely to present a risk of delay in treatment due to the late referral of instruction for jaundiced infants. The WHO and IPS tend to have overlapping sets of recommendations. The WHO guidelines tend to be simpler, have a lower threshold of intervention and have a straightforward set of treatment intervention recommendations compared to the IPS. Evaluation of the applicability of the WHO and IPS guidelines in the field, and unity of recommendations, is needed to prevent confusion in the management of yellow babies in Indonesia.

## Data availability

### Underlying data

• The Indonesian Pediatric Society (IPS) guidelines assessed in this study are freely available (in Indonesian) from Ikatan Dokter Anak Indonesia (IDAI), available here: https://www.idai.or.id/professional-resources/pedoman-konsensus/pedoman-pelayanan-medis-2
• The Ministry of Health (MoH) guidelines assessed in this study are freely available (in Indonesian) from Kementerian Kesehatan RI, available here: http://ambariani.staff.gunadarma.ac.id/Downloads/files/60594/Buku-Saku-Pelayanan-Kesehatan-Neonatal-Esensial.pdf
• World Health Organization (WHO) guidelines assessed in this study are freely available (in English), from WHO here: https://apps.who.int/iris/bitstream/10665/81170/1/9789241548373_eng.pdf

The authors are also in the process of translating the Indonesian guidelines into English, but these were not finished at the time of publication. Interested readers should contact the corresponding author with any translation requests.
