# Supplementary material for: A review of existing neonatal hyperbilirubinemia guidelines in Indonesia
Source: F1000Res. 2023 Nov 13;11:1534. Originally published 2022 Dec 19. [Version 2] doi: 10.12688/f1000research.110550.2 (PMC10682606; doi:10.12688/f1000research.110550.2)
Supplement: Peer review history

# Reviewer report: Reviewer response for version 2

**DOI:** 10.5256/f1000research.158223.r222675

**Published:** 2023-11-27

**Authors:** Ramesh Vidavalur

**Affiliations:** 1 Cayuga Medical Center, Ithaca, New York, USA

Authors have included appropriate revisions as per reviewer comments. Thank you.

Is the work clearly and accurately presented and does it cite the current literature?

Yes

If applicable, is the statistical analysis and its interpretation appropriate?

Not applicable

Are all the source data underlying the results available to ensure full reproducibility?

Yes

Is the study design appropriate and is the work technically sound?

Yes

Are the conclusions drawn adequately supported by the results?

Yes

Are sufficient details of methods and analysis provided to allow replication by others?

Yes

Reviewer Expertise:

Neonatology, Cost benefit analysis, Health policy

I confirm that I have read this submission and believe that I have an appropriate level of expertise to confirm that it is of an acceptable scientific standard.

# Reviewer report: Reviewer response for version 2

**DOI:** 10.5256/f1000research.158223.r222676

**Published:** 2023-11-27

**Authors:** Thor Willy Ruud Hansen

**Affiliations:** 1 Faculty of Medicine, University of Oslo, Oslo, Norway

It looks like the authors have taken my comments into appropriate consideration in their revision of the manuscript. Thus, at this point I have no objections to the indexing of this MS.

Is the work clearly and accurately presented and does it cite the current literature?

Yes

If applicable, is the statistical analysis and its interpretation appropriate?

Not applicable

Are all the source data underlying the results available to ensure full reproducibility?

No source data required

Is the study design appropriate and is the work technically sound?

Partly

Are the conclusions drawn adequately supported by the results?

Yes

Are sufficient details of methods and analysis provided to allow replication by others?

Partly

Reviewer Expertise:

NA

I confirm that I have read this submission and believe that I have an appropriate level of expertise to confirm that it is of an acceptable scientific standard.

# Reviewer report: Reviewer response for version 1

**DOI:** 10.5256/f1000research.122172.r193746

**Published:** 2023-08-23

**Authors:** Ramesh Vidavalur

**Affiliations:** 1 Cayuga Medical Center, Ithaca, New York, USA

This manuscript compares three existing approaches for detection, management and follow up of neonatal hyperbilirubinemia in Indonesia. There were notable differences between these guidelines that impacts care of newborns. The authors point out the pitfalls of generalized guidelines outlined by Ministry of health and WHO recommend pre-discharge bilirubin screening with careful evaluation of risk factors. I recommend discussing recent 2022 AAP guidelines in the manuscript and how the authors can adapt these guidelines to country-specific context. My suggestion for the authors is to develop and produce a uniform guideline and include it in the manuscript for general practitioners in the country.

Is the work clearly and accurately presented and does it cite the current literature?

Yes

If applicable, is the statistical analysis and its interpretation appropriate?

Not applicable

Are all the source data underlying the results available to ensure full reproducibility?

Yes

Is the study design appropriate and is the work technically sound?

Yes

Are the conclusions drawn adequately supported by the results?

Yes

Are sufficient details of methods and analysis provided to allow replication by others?

Yes

Reviewer Expertise:

Neonatology, Cost benefit analysis, Health policy

I confirm that I have read this submission and believe that I have an appropriate level of expertise to confirm that it is of an acceptable scientific standard, however I have significant reservations, as outlined above.

# Response

**Published:** 2023-10-28

**Authors:** Mahendra Tri Arif Sampurna

**Affiliations:** Faculty of Medicine Universitas Airlangga, Indonesia

We are very thankful to Assistant Professor Ramesh Vidavalur for your availability and willingness to help us to become our reviewer on our manuscript. We have revised our manuscript based point-on-point revision suggested.

 1) I recommend discussing recent 2022 AAP guidelines in the manuscript and how the authors can adapt these guidelines to country-specific context. My suggestion for the authors is to develop and produce a uniform guideline and include it in the manuscript for general practitioners in the country.

Response: Thank you for your valuable suggestion, we very much agree with you and we have added the point and relevance of our included guideline with the new 2022 AAP guideline in the manuscript.

# Reviewer report: Reviewer response for version 1

**DOI:** 10.5256/f1000research.122172.r168807

**Published:** 2023-04-14

**Authors:** Thor Willy Ruud Hansen

**Affiliations:** 1 Faculty of Medicine, University of Oslo, Oslo, Norway

I appreciate the opportunity to read this important paper. However, it is difficult to review this paper in some regards, as it mainly consists of an assessment of three different guidelines. This assessment seems to be somewhat subjective, and it is possible that this was the only way to perform this work. However, this makes a critique also somewhat subjective. Thus, I am going to approach this by sharing some thoughts with the authors, with the understanding that the authors may have valid disagreements with my thoughts.

 Clearly, given the data presented on the incidence of kernicterus in Indonesia, neonatal jaundice (NJ) is a very important neonatal health issue in that country, and finding ways to combat this problem is of paramount importance. 

 It is clearly a challenge that 3 different guidelines for NJ are in use by different categories of care providers, 2 of these are of national original origin (IPS and MoH), while the WHO guidelines are of international origin. The WHO guidelines are in a sense "generic", and while they are focused on the situation in LIMCs, they cannot account for specifics in the health care delivery systems in each individual country. Thus, the knowledge necessary to craft guidelines which are tailored to the specifics of health care in Indonesia clearly resides with the professional community in Indonesia. I don't know anything about the "balance of power" between the MoH and the IPS in Indonesia. Thus, my thoughts on the path forward may be naive, based as they are on my life and practice in a small country with just over 5 million inhabitants and a well equipped and organized healthcare system. However, with that caveat I would think of the MoH as the repository of knowledge (and power) as regards the health care system at large, including its organization and distribution of resources, while the IPS is the repository of professional knowledge on how to manage newborn infants with NJ. It seems very clear to me that as the authors' goal is to reduce the incidence of kernicterus, this problem must be approached from both the public health perspective and the individual perspective. Thus, my starting point on the way forward would NOT be to produce a new guideline, but to have the IPS contact the MoH and organize a conference to i) discuss the problem, ii) share thoughts on how the challenges may be approached both from the organizational perspective, the health care providers' perspective, and the social perspective. A possible way forward might then be to establish a working group with representatives both form the MoH, the IPS, and the nurses' and midwives' organizations. Thus, to me this seems to be a problem that not only involves diagnostic and therapeutic methods and technologies, but also the training (and re-training) of health care providers, as well as efforts to identify the roadblocks in access to care, which are likely to be determinants for failure in many individual cases. 

 The authors refer to the AAP guidelines, which are very well researched and presented. However, the AAP guidelines are directed to the US health care system, and it is very likely that the challenges to health care delivery in Indonesia are different from those in the USA. Indeed, the authors refer to this on p.3. Thus, the way forward must go through the identification of the things that are characteristic of, and possibly also specific to, the Indonesian reality. While the thoughts that I have outlined above are clearly not a "quick and easy fix", I think a simple focus on creating new charts and procedures may fail if not properly adapted to health care in Indonesia.

 These were my comments of a more general nature. There are some details in the manuscript that call for a more detailed proof-reading. I notice that the authors have availed themselves of professional proofing and language editing services, but several glitches have never the less occurred. 

 As far as the key words, I suggest that the authors look at key words used in recent papers on NJ and clinical practice. Thus, I think that "guidelines" are more likely to be used than "recommendations", and "jaundice, neonatal" more commonly used than "icterus". Adding the word "infant OR newborn" may be helpful for those looking for articles on the subject.

 The first paragraph under the heading "Source data" on p.3 is not well expressed, and would benefit from revision. 

 In figures 1 and 2, I would suggest amending "threshold term and ..." to "threshold for term and...".

 Under the heading "Monitoring" I am puzzled at "2-24" days - should it be "2-4" days?

 In table 1, under IPS, I suggest "increased enterohepatic circulation" and "defective uptake", Under WHO, NJ lasting more than 14 days may be "breast milk associated jaundice", which may not necessarily be "non-physiological, but I recognize that this may be a metter of judgment. 

 In table 2, the heading should be "Set of recommendations...". Under IPS, I believe that "antiripsin" should be "antitrypsin". IPS point 7, I think "hematomas" should perhaps be in the list. Under MoH, should perhaps "sugar" be "blood sugar"? Under WHO, I believe "venereal disease research laboratory" is commonly just written VDRL.

 In table 3, I think at "in the high risk area" needs a reference to the nomogram to which you refer. Whether the Bhutani nomogram is applicable in Indonesia may be doubtful, but there may be references there of which I am not aware.

 On p.4, is it possible that something is missing from "in the NICE guideline conclude from..."? It doesn't quite make sense to me.

 On p.5, "a uniformed guideline" should be "a uniform guideline".

 Further down, what is "low PT machines"? Further down again: "we recommend to develop a new unity guidelines" would better be "we recommend developing a new uniform guideline".

Is the work clearly and accurately presented and does it cite the current literature?

Yes

If applicable, is the statistical analysis and its interpretation appropriate?

Not applicable

Are all the source data underlying the results available to ensure full reproducibility?

No source data required

Is the study design appropriate and is the work technically sound?

Partly

Are the conclusions drawn adequately supported by the results?

Yes

Are sufficient details of methods and analysis provided to allow replication by others?

Partly

Reviewer Expertise:

Neurotoxicology; Neonatal jaundice; kernicterus

I confirm that I have read this submission and believe that I have an appropriate level of expertise to confirm that it is of an acceptable scientific standard, however I have significant reservations, as outlined above.

# Response

**Published:** 2023-10-28

**Authors:** Mahendra Tri Arif Sampurna

**Affiliations:** Faculty of Medicine Universitas Airlangga, Indonesia

We are very thankful to Professor Thor Willy Ruud Hansen for your availability and willingness to help us to become our reviewer on our manuscript. We have revised our manuscript based point-on-point revision suggested.

 1) As far as the key words, I suggest that the authors look at key words used in recent papers on NJ and clinical practice. Thus, I think that "guidelines" are more likely to be used than "recommendations", and "jaundice, neonatal" more commonly used than "icterus". Adding the word "infant OR newborn" may be helpful for those looking for articles on the subject.

Response: Thank you for your valuable suggestion, we agree with you and have revised the phrase in the manuscript.

                

 2) The first paragraph under the heading "Source data" on p.3 is not well expressed, and would benefit from revision.

Response: Thank you for your valuable suggestion, we have revised this paragraph.

 3) In figures 1 and 2, I would suggest amending "threshold term and ..." to "threshold for term and...".

Response: Thank you for your valuable suggestion, we have revised the figures.

 4) Under the heading "Monitoring" I am puzzled at "2-24" days - should it be "2-4" days?

Response: Thank you for your valuable question, the correct is 2-4 days. We have revised this in the manuscript.

 5) In table 1, under IPS, I suggest "increased enterohepatic circulation" and "defective uptake", Under WHO, NJ lasting more than 14 days may be "breast milk associated jaundice", which may not necessarily be "non-physiological, but I recognize that this may be a metter of judgment.

Response: Thank you for your valuable suggestion, we agree with you and have revised the phrase in the manuscript. However, we keep “non-physiological” because we adhere to the phrase from the source (WHO).

 6) In table 2, the heading should be "Set of recommendations...". Under IPS, I believe that "antiripsin" should be "antitrypsin". IPS point 7, I think "hematomas" should perhaps be in the list. Under MoH, should perhaps "sugar" be "blood sugar"? Under WHO, I believe "venereal disease research laboratory" is commonly just written VDRL.        

Response: Thank you for your valuable suggestion, we agree with you and have revised the phrase in the manuscript.

 7) In table 3, I think at "in the high risk area" needs a reference to the nomogram to which you refer. Whether the Bhutani nomogram is applicable in Indonesia may be doubtful, but there may be references there of which I am not aware.

Response: Thank you for your valuable suggestion, we used the Bhutani nomogram, therefore we agree with you to revise the phrase and add information about the nomogram used.

 8) On p.4, is it possible that something is missing from "in the NICE guideline conclude from..."? It doesn't quite make sense to me.

Response: Thank you for your valuable suggestion, we acknowledge our mistake and have revised the manuscript.

 9) On p.5, "a uniformed guideline" should be "a uniform guideline".

Response: Thank you for your valuable suggestion, we acknowledge our mistake and have revised the manuscript.

 10) Further down, what is "low PT machines"?

Response: Thank you for your valuable question, what we mean is lower phototherapy device, we have revised this sentence in the manuscript.

 11) Further down again: "we recommend to develop a new unity guidelines" would better be "we recommend developing a new uniform guideline".

Response: Thank you for your valuable suggestion, we very much agree with you and have revised the manuscript.
